# The Genetic Architecture of Maize (*Zea mays* L.) Kernel Weight Determination

**DOI:** 10.1534/g3.114.013243

**Published:** 2014-09-01

**Authors:** Santiago Alvarez Prado, César G. López, M. Lynn Senior, Lucas Borrás

**Affiliations:** * Facultad de Ciencias Agrarias, Universidad Nacional de Rosario, Zavalla S2125ZAA, Prov. de Santa Fe, Argentina; † Facultad de Ciencias Agrarias, Universidad de Lomas de Zamora, Lavallol 1836, Prov. de Buenos Aires, Argentina; ‡ Syngenta Seeds Inc., Research Triangle Park, North Carolina 27709

**Keywords:** kernel weight, kernel growth rate, grain-filling duration, genetic background effects, complex traits, Multiparent Advanced Generation Inter-Cross (MAGIC), multiparental populations, MPP

## Abstract

Individual kernel weight is an important trait for maize yield determination. We have identified genomic regions controlling this trait by using the B73xMo17 population; however, the effect of genetic background on control of this complex trait and its physiological components is not yet known. The objective of this study was to understand how genetic background affected our previous results. Two nested stable recombinant inbred line populations (N209xMo17 and R18xMo17) were designed for this purpose. A total of 408 recombinant inbred lines were genotyped and phenotyped at two environments for kernel weight and five other traits related to kernel growth and development. All traits showed very high and significant (*P* < 0.001) phenotypic variability and medium-to-high heritability (0.60−0.90). When N209xMo17 and R18xMo17 were analyzed separately, a total of 23 environmentally stable quantitative trait loci (QTL) and five epistatic interactions were detected for N209xMo17. For R18xMo17, 59 environmentally stable QTL and 17 epistatic interactions were detected. A joint analysis detected 14 stable QTL regardless of the genetic background. Between 57 and 83% of detected QTL were population specific, denoting medium-to-high genetic background effects. This percentage was dependent on the trait. A meta-analysis including our previous B73xMo17 results identified five relevant genomic regions deserving further characterization. In summary, our grain filling traits were dominated by small additive QTL with several epistatic and few environmental interactions and medium-to-high genetic background effects. This study demonstrates that the number of detected QTL and additive effects for different physiologically related grain filling traits need to be understood relative to the specific germplasm.

Improving yield is the main objective of breeding programs in grain crops. For the majority of the twentieth century, maize grain yield was improved by applying selection techniques over phenotypic measurements (Hallauer and Miranda 1988). Since 1980, detection of quantitative trait loci (QTL) combining phenotypic information with molecular marker data has received considerable attention (Bernardo 2008). A large number of studies identifying molecular markers linked to QTL involved in the inheritance of agronomically relevant traits such as grain yield have been described (Bernardo 2008).

Grain yield is the final outcome of many processes occurring throughout the growing season. One avenue to investigating the genetic basis of yield is to dissect it into individual yield components and then search for their specific genetic basis (Slafer 2003). Maize grain yield can be described as a function of the number of harvested kernels and their individual weight. Of these two components, kernel number is usually the one that explains most variation; however, both components affect final yield (Borrás and Gambín 2010). Kernel weight (KW) is a highly heritable trait (Sadras 2007; Alvarez Prado *et al.* 2013a), varying markedly among genotypes (Reddy and Daynard 1983).

The determination of KW is generally described by traits related to dry matter and water content accumulation (Schnyder and Baum 1992; Borrás *et al.* 2003; Bingham *et al.* 2007; Rondanini *et al.* 2007) and is commonly divided into three phases: the lag phase, the effective grain-filling period, and the maturation drying phase (Figure 1; Bewley and Black 1985). The lag phase is a period of active cell division characterized by water content increases with almost no dry matter accumulation. The effective grain-filling period is characterized by rapid dry matter accumulation at a constant rate resulting from the deposition of reserves. Most genotypic differences in KW are related to changes in the kernel growth rate (KGR) around this period. KGR is very dependent on the sink capacity established early in grain filling and can be estimated with the kernel maximum water content (MWC; Figure 1D). Moisture concentration (MC) within kernels is reduced throughout grain filling (Figure 1C). At a particular critical MC biomass deposition stops and total grain-filling duration (GFD) is established. This moment is known as physiological maturity (Shaw and Loomis 1950). As such, GFD depends on the rate of kernel desiccation (KDR) and the MC that each specific genotype attains physiological maturity (MCPM; Figure 1D). All these traits vary among exotic and elite germplasm (Borrás *et al.* 2009), and we are interested in studying their genetic basis.

**Figure 1:**
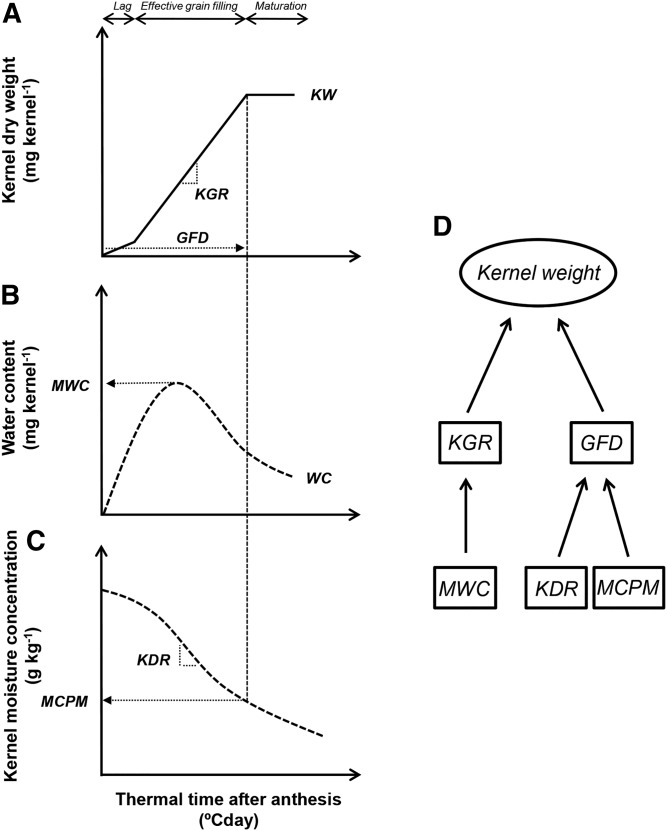
Schematic figure describing phenotypic grain-filling traits of interest: (A) kernel weight (KW), kernel growth rate (KGR), and grain-filling duration (GFD); (B) maximum water content (MWC); (C) moisture concentration at physiological maturity (MCPM) and kernel desiccation rate during the effective grain-filling period (KDR); and (D) conceptual representation of trait hierarchy and correlated physiological mechanisms. See the section *Materials and Methods* for details regarding the measurement of each specific trait. Figure was adapted from Alvarez Prado *et al.*, (2013b).

Several studies on QTL mapping for maize KW have been conducted, and inconsistent results in terms of localization and effect size were obtained (Schön *et al.* 1994; Austin and Lee 1996, 1998; Frova *et al.* 1999). The lack of consistency could be related to the complexity of the trait, needing further dissection into simpler components. KW is commonly dissected in its physiological components KGR and GFD. These traits are governed by different physiological mechanisms (Borrás and Gambín 2010). They also are genetically independent traits as genomic regions associated with their determination do not colocalize (Alvarez Prado *et al.* 2013b). Depending on the specific germplasm used at each study, KW variability could be related to differences in GFD or KGR only (Figure 1D). These differential mechanisms behind genetic differences in KW can generate inconsistent QTL localizations.

Most previous studies dealing with QTL and KW determination have been conducted using different individual biparental populations. At these populations, only two alleles at any given locus are simultaneously tested, without representing the genetic variability of the species (Holland 2007). Linkage mapping based on biparental populations can only identify QTL from the phenotypic diversity generated from the controlled cross. Use of multiple-cross mating designs sharing the same (Yu *et al.* 2008; Li *et al.* 2011) or different parents (Kraakman *et al.* 2004; Blanc *et al.* 2006; Verhoeven *et al.* 2006) enable higher power and resolution through joint linkage and association analyses. Statistical methods are currently available to correctly analyze connected populations (Jannink 2007; Malosetti *et al.* 2007; Li *et al.* 2011).

The objective of our study was to unravel the genetic architecture of maize KW determination by using a multiple-parental population generated from contrasting parental lines for KW and its physiological components. We were specifically interested in using a connected population because our final trait of interest (KW) is determined through different physiological mechanisms. Testing a larger range of variability than commonly explored in biparental populations seemed critical for our needs.

## Materials and Methods

### Plant material and experimental design

Lines Mo17 (PI 558532), N209 (PI 595366), and R18 (PI 340856) were selected as parental lines for recombinant inbred line (RIL) population development. Selection of parental lines was based on their variability for grain-filling patterns (Borrás *et al.* 2009). We used Mo17 as the common parent because we also had available the RIL population B73xMo17 (IBM Syn4).

Mo17 is a yellow endosperm maize inbred with a red cob developed from the cross CI187-2xC103 (Zuber 1973). This inbred has a KW of c.a. 280 mg kernel^−1^, determined by a high KGR and a moderate GFD (Borrás *et al.* 2009). N209 is a yellow dent maize inbred developed directly from the NSSI (6), the sixth cycle of *per se* selection in the Nebraska Stiff Stalk Synthetic (Kaeppler *et al.* 1997). This inbred shows an average KW (c.a. 260 mg kernel^−1^) determined by a long GFD and a moderate KGR. Inbred R18 was derived from the Supergold population (www.ars-grin.gov) with red pericarp. This inbred has a very small KW (c.a. 100 mg kernel^−1^) due to a reduced KGR and GFD.

F_1_ crosses R18xMo17 and N209xMo17 were made at the Agronomy Department of Iowa State University, Ames, Iowa, during 2006/07 (Table 1) with parental seeds obtained from the USDA (www.ars-grin.gov). A single F_1_ plant was used to generate the F_2_ at Brunner Farm, Iowa State University, during 2007 (Table 1). These F_2_ were advanced to the F_6_ generation by single-seed descent and multiplied after. Generations were done in the United States and Argentina (Table 1). For this study, we used 204 RILs from each population (R18xMo17 and N209xMo17). Depending upon availability 10−15K per RIL is available for free by contacting Dr. L. Borrás (lborras@unr.edu.ar). Shipping costs must be covered by the requester.

**Table 1:** General description of the N209xMo17 and R18xMo17 mapping population development

Generation	Season	Growth Conditions	Location
F_1_	2006/07	Greenhouse	Ames, Iowa (USA)
F_2_	2007	Field	Ames, Iowa (USA)
F_3_	2007/08	Field	Buenos Aires (ARG)
F_4_	2008	Field	Slater, Iowa (USA)
F_5_	2008/09	Field	Santa Isabel, Santa Fe (ARG)
F_6_	2009/10	Field	Venado Tuerto, Santa Fe (ARG)

ARG, Argentina.

Field experiments were conducted during 2010/2011 (hereafter 2010) and 2011/2012 (hereafter 2011) growing seasons at the Campo Experimental Villarino, Facultad de Ciencias Agrarias, Universidad Nacional de Rosario, at Zavalla, Argentina. Each experiment was arranged in a randomized complete block design with three replicates. Planting was October 4, 2010, and September 22, 2011. A stand density of 7 pl m^−2^ was used for both experiments. Plots were overplanted and thinned at V2 and were one row 0.52 m apart and 5.5 m long.

Experiments were conducted without water limitations using a sprinkler irrigation system. Pests and diseases were controlled by spraying commercially recommended fungicides and insecticides, and weeds were periodically removed by hand.

### Phenotypic measurements and analysis

Kernel dry matter and water content were measured throughout kernel development beginning 15 d after each plot reached anthesis and until harvest maturity (15% kernel MC) following procedures described in Borrás *et al.* (2003). To summarize, one plant per plot was sampled every 4 or 5 d between 07:00 and 10:00 am. The entire ear with surrounding husks was immediately enclosed in an airtight plastic bag and transported to the lab. Kernels were removed from the ear at floret positions 10−15 from the bottom of the rachis within a humidified box. Ten kernels per ear were sampled on each date. Fresh weight was measured immediately after sampling, and kernel dry weight was determined after drying samples at 70° for at least 96 hr. Fresh and dry weight were used to calculate kernel water content and kernel MC.

KGR and GFD were determined for each genotype x replicate combination by fitting a bilinear model (Equation [1] and [2]) as in Borrás *et al.* (2009) (Figure 1A):

KW=a+b TT for TT≤c (1)

KW=a+bc  for TT>c (2)

where TT is the number of heat units after pollination (°Cday), *a* is the *y*-intercept (mg kernel^−1^), *b* is the KGR during the effective grain-filling period (mg °Cday^-1^), and *c* is the GFD (°Cday) (Figure 1A). Heat units were calculated using 0° as base temperature (Muchow 1990; Borrás *et al.* 2009). Mean daily air temperature was registered at a weather station located approximately 150 m from the experimental plots.

Kernel MWC was determined for each genotype x replicate combination by fitting a curvilinear model (Equation [3]) as in Borrás *et al.* (2009) (Figure 1B):

$$\mathrm{WC}=d+e\;\mathrm{TT}+fTT^{1.5}+g\;TT^{2}$$ (3)

where WC is kernel water content and *d*, *e*, *f*, and *g* are model parameters.

Kernel MCPM was determined using a bilinear model relating kernel dry weight and kernel MC data (Equation [4] and [5]) following Borrás *et al.* (2009):

KW=h−iMC for MC≥j (4)

KW=h−i j  for MC<j (5)

where MC is moisture concentration (%), *h* is the *y*-intercept (mg kernel^−1^), *i* is the rate of kernel MC decline during grain filling (mg kernel^−1^ [%]^−1^), and *j* is the MCPM (%). This model was fitted for each genotype x replicate combination.

KDR was determined using a linear regression model fitted for each genotype x replicate combination relating kernel MC and thermal time from pollination to physiological maturity:

MC=k+l TT (6)

where MC is kernel MC (g kg^−1^), *k* is the *y*-intercept (g kg^−1^), and *l* is the KDR (g kg^−1^ °Cday^−1^).

All curves were fitted using the iterative optimization technique of GraphPad Prism V5.0 (Raduschev 2007).

Phenotypic variance was partitioned into genetic and environmental components by using a mixed model for each population and trait separately. The model included environments (years), blocks nested within environments, genotypes and genotype x environment interaction. All model factors were considered as random. Proc MIXED using REML method from SAS statistical package was used for the analysis (SAS Institute 1999).

Broad sense heritability of each trait was calculated on a mean basis as:

$$H^{2}=\sigma_{G}^{2}/\left[\sigma_{G}^{2}+\left(\sigma_{\mathrm{GE}}^{2}/\eta \right)+\sigma_{e}^{2}/r\eta \right]$$ (7)

where σ^2^_G_ is the genotypic variance, σ^2^_GE_ is the genotype x environment variance, σ^2^_e_ is the plot residual variance, and η and *r* are the number of environments and replication plots, respectively (Holland *et al.* 2003).

For QTL analysis, phenotypic data were analyzed by using a different mixed model approach following Malosetti *et al.* (2008). The multitrait multienvironment model was performed on each population separately where the data set consisted of *I* genotypes evaluated in *J* environments with measurements on *K* traits (in our study, *I* = 204 for each population, *J* = 2, and *K* = 6). Define an *N* x 1 vector “y,” with *N* = *IJK* that contains all the observations sorted by trait within environment for each population. Given that the interest is in the genetic variation within the population rather than the genotypes themselves, we assumed genotypes to be random. Trait-environment (TE) combinations and blocks nested within TE combination were considered as fixed. The mixed linear model for multitrait multienvironment data were as follows:

y=X β+Z u+ε (8)

where *y* is the vector of phenotypic observations, *β* is a vector of fixed effects due to the TE combination and blocks within TE, while *u* and* ε* are the vectors of random effects due genotypes and residuals, respectively. *X* and *Z* are incidence matrices of 1s and 0s associated to fixed and random effects, respectively. Vector *β* contains the trait means within environments across genotypes and the blocks means within TE combination. Vector *u* denotes the random genotypic effects per TE combination. Random genetic effects were assumed to be normally distributed *u* ~ N (0, *G*), being the *G* matrix a block diagonal with blocks of 12 × 12 variance-covariance parameters. Finally, *ε* is a vector of non-genetic residuals associated with each observation and normally distributed *ε~* N (0, *R*) being *R* the residual variance (*I*σ^2^_ijk_). The phenotypic (co)variance was:

$$V\left(y\right)=ZGZ^{\prime }+R$$ (9)

Different variance-covariance structures for the *G* matrix were assumed to select the most suitable and parsimonious for our data set. All assumed structure models considered the factorial combination of traits and environments, interpreting each TE combination as a trait by itself. Evaluated structures used different parameter number for fitting data. Thus, the choice of the best model for our data was based on a goodness of fit criterion such as the Bayesian Information Criterion (Schwarz 1978). Compound symmetry model was selected as the best model for both populations. Considering this variance-covariance structure, best linear unbiased predictor of each genotype in each environment was estimated for reducing uncontrolled trait variation for QTL mapping (Alvarez Prado *et al.* 2013b).

### Genetic map construction and QTL analysis

The 408 stable recombinant inbred lines (RILs) and the parental lines of each population were genetically characterized with 500 public single-nucleotide polymorphisms. A total of 127 and 130 single-nucleotide polymorphisms (Supporting Information, File S1) were polymorphic and showed no segregation distortion for populations R18xMo17 and N209xMo17, respectively. Linkage analysis was carried out using JoinMap v4 (Van Ooijen 2006). Individual genetic maps for each population and a consensus genetic map were constructed. Map distances were computed with the Haldane mapping function (Haldane 1919).

#### Statistical models for QTL analysis at individual populations:

For individual populations, a QTL analysis was carried out using the inclusive composite interval mapping (ICIM) proposed by Li *et al.* (2007) using MET option from QTL IciMapping software (available from www.isbreeding.net). As composite interval mapping, ICIM method uses a linear regression model:

$$y_{i}=b_{0}+\sum_{j=1}^{m+1}b_{j}x_{ij}+e_{i},$$ (10)

where *y_i_* is the trait value of the *i*th individual in the mapping population; *b*_0_ is the overall mean of the model; *x_ij_* is a dummy variable for the genotype of the *i*th individual at the *j*th marker; *b_j_* is the regression coefficient of the phenotype on the *j*th marker conditional on all other markers; and *e_i_* is the residual error that is assumed normally distributed.

When building the model (10), ICIM uses all marker information by selecting the most important markers and setting to zero the unselected ones through a stepwise regression. In order to avoid over fitting a probability of 0.0001 was used (Li *et al.* 2008). Thus, for a testing position in a specific interval (*k*, *k* + 1) the phenotypic values from model (10) are adjusted by:

$$\Delta y_{i}=y_{i}-\sum_{j\neq k,\;k+1}{\hat{b}}_{j}x_{ij}+e_{i},$$ (11)

where ${\hat{b}}_{j}$ is the estimate of *b_j_* in model (10). Phenotypic values were adjusted by all markers retained in the regression equation except the two markers flanking the current interval. The adjusted phenotype (*Δy_i_*) contains the position and additive effect information of the QTL in the current testing position and excludes the influence of the QTL located on other interval or other chromosomes (Wang *et al.* 2012). A logarithm of odds (LOD) score of 2.5 and a step size of 2 cM were used for QTL analysis.

Digenic epistasis of QTL was estimated using the linear regression model developed by Li *et al.* (2008) in QTL IciMapping software:

$$y_{i}=b_{0}+\sum_{j=1j<k}^{m+1}b_{j}x_{ij}+\sum b_{jk}x_{ij}x_{ik}+e_{i},$$ (12)

where *y_i_* is the phenotypic value of the trait for the *i*th individual in the mapping population; *b_0_* is the overall mean; *x_ij_* is a dummy variable for the genotype of the *i*th individual at the *j*th marker; *b_j_* is the partial regression coefficient of the phenotype on the *j*th marker variable; *b_jk_* is the partial regression coefficient of the phenotype on the multiplication variable of the *j*th and *k*th markers; and *e_i_* is the residual error that is assumed normally distributed.

A two-stage stepwise regression strategy was adopted for parameter estimation. First, significant markers were selected. Then stepwise regression with a probability of 0.0001 was applied to the residuals from the first stage to select significant markers pairs and estimate their effects in the model (Li *et al.* 2008):

$$\Delta y_{i}=y_{i}-\sum_{r\neq j,j+1,k,k+1}{\hat{b}}_{j}x_{ij}-\sum_{\begin{array}{l} r\neq j,j+1\\ s\neq k,k+1 \end{array}}{\hat{b}}_{rs}x_{ir}x_{is}+e_{i},$$ (13)

where ${\hat{b}}_{r}$ and ${\hat{b}}_{rs}$ are the estimates of *b_r_* and *b_rs_* in model (12), respectively. The adjusted phenotype *Δy_i_* thus obtained contains the information of QTL in the two testing intervals including two positions, two additive effects of individual QTL, and one epistatic effect between the two QTL. A LOD score of 5 and a step size of 10 cM for improving detection accuracy were used for epistasis detection (Zhang *et al.* 2012).

#### Statistical models for joint QTL mapping:

For statistical analysis of combined populations we used joint ICIM, developed by Li *et al.* (2011). The joint ICIM method is similar to model (11) but includes a population effect term:

$$\Delta y_{if}=y_{if}-\alpha_{if}u_{f}\sum_{j\neq k,\;k+1}{\hat{b}}_{j}x_{ifj},$$ (14)

where *u_f_* is the background effect of each founder with the common parent for the *f*th population and *α_if_* is a dummy variable for the genotype of the *i*th individual at the *f*th population. Because equations (11) and (14) differed only in the background effect (*u_f_*), this was estimated as the proportion of QTL number difference between QTL analysis of individual populations and that of joint populations.

#### Meta QTL analysis:

To study QTL congruency a meta QTL analysis including results from the two evaluated populations and the IBM (B73xMo17) Syn-4 population (Alvarez Prado *et al.* 2013b) was performed using Biomercator v3 (Sosnowski *et al.* 2012). We conducted a meta QTL analysis instead of a joint QTL analysis because evaluated populations shared only one of the two tested environments and because B73xMo17 shared very few molecular markers with the other two populations. Meta QTL analysis uses a two-stage procedure to integrate multiple independent QTL experiments. The first step consisted of integrating individual genetic maps into a consensus map. For connecting maps, the procedure needed at least two common markers per chromosome between maps. In those cases in which there were fewer than two common markers per chromosome between two maps, marker physical positions (http://www.maizegdb.org) were compared and those markers showing to be in tight linkage (overlapped positions) were considered as synonyms for the purpose of the meta QTL analysis. Genetic map from N209xMo17 was used as reference map because it had more markers in common with remaining populations.

The second step consisted on projecting QTL from individual studies on the consensus map. By assuming there are a finite number of true QTL locations, the meta QTL algorithm performed a clustering approach based on a Gaussian-mixture distribution to both classify the observed QTL and estimate its position (Veyrieras *et al.* 2007). We later continued testing all possible QTL combinations and then choosing the one that maximizes a penalized log-likelihood. To select the number of meta QTL, the software provides five different criteria for model choice (Veyrieras *et al.* 2007). In this study, Akaike Information Criterion (Akaike 1974) was used to select final number of meta QTL.

## Results

### Phenotypic results

Phenotypes from both RIL populations showed narrow differences in time to silking. The accumulated thermal time from planting to the first flowering line and to the last flowering line were 951 to 1269 °Cday (representing 19 d) and 985 to 1286 °Cday (18 d) for R18xMo17 and N209xMo17, respectively, in 2010. The accumulated thermal times were from 1107 to 1428 °Cday (20 d) and 1107 to 1444 °Cday (21 d) for R18xMo17 and N209xMo17, respectively, in 2011. Most important, phenotypic variation in time to silking was not associated with any grain-filling trait (*P* > 0.05).

The coefficient of variation for all phenotyped traits ranged from 7 to 13% (Table 2), allowing a precise estimation of variance components and heritability. Designed populations explored a wide range of phenotypic values for all grain-filling traits (Table 2). Approximately 73 and 49% of KW variation in R18xMo17 and N209xMo17, respectively, was genotypic variation (Table 2).

**Table 2:** Kernel weight, kernel growth rate, grain-filling duration, maximum water content, kernel desiccation rate, and moisture concentration at physiological maturity for the parental lines and RILs from R18xMo17 and N209xMo17 populations growing at two environments (2010 and 2011)

Population	Environment	Genotype	Kernel weight, mg kernel^−1^	Kernel growth rate, mg °Cday^−1^	Grain- filling duration, °Cday	Maximum water content, mg kernel^−1^	Kernel desiccation rate, g kg^−1^ °Cday^−1^	Moisture concentration at maturity, %
R18xMo17	2010	R18	107	0.164	913	63	0.719	34.3
		Mo17	260	0.377	962	259	0.737	42.3
		Mean	160	0.264	875	117	0.737	34.5
		Min.	112	0.182	770	76	0.579	28.6
		Max.	212	0.410	992	176	0.959	42.8
	2011	R18	132	0.236	679	66	0.740	29.5
		Mo17	271	0.401	963	243	0.665	39.3
		Mean	170	0.270	917	123	0.734	33.3
		Min.	111	0.183	750	81	0.613	24.8
		Max.	249	0.376	1035	180	0.936	42.9
	CV (%)		7.2	13.4	7.3	8.0	10.0	11.8
	H^2^		0.92 ± 0.01	0.80 ± 0.03	0.62 ± 0.01	0.92 ± 0.01	0.60 ± 0.06	0.66 ± 0.05
	Genotype (G)		664.8	1617.4	0.0014	525.3	0.0027	6.1
	Environment (E)		48.2	1408.7	0.0000	5.3	0.0000	1.5
	G x E		50.3	521.1	0.0003	45.1	0.0007	2.7
	Residual		146.3	4248.3	0.0012	93.1	0.0056	15.6
N209xMo17	2010	N209	225	0.268	1113	171	0.643	34.9
		Mo17	260	0.377	962	245	0.736	43.4
		Mean	238	0.362	983	210	0.669	39.4
		Min.	157	0.278	774	154	0.546	28.7
		Max.	301	0.513	1173	290	0.769	51.2
	2011	N209	244	0.304	1096	188	0.585	36.7
		Mo17	271	0.401	963	243	0.665	39.3
		Mean	249	0.352	1028	220	0.628	39.2
		Min.	171	0.276	752	165	0.534	31.7
		Max.	310	0.520	1182	283	0.764	48.9
	CV (%)		7.9	12.6	7.8	8.3	9.3	11.0
	H^2^		0.77 ± 0.04	0.61 ± 0.07	0.37 ± 0.10	0.88 ± 0.02	0.66 ± 0.05	0.54 ± 0.07
	Genotype (G)		731.8	1651.1	0.0013	802.4	0.0015	9.7
	Environment (E)		81.8	1322.4	0.0000	36.8	0.0005	0.3
	G x E		288.4	3189.2	0.0009	91.8	0.0003	9.4
	Residual		388.4	6342.9	0.0019	325.1	0.0035	18.3

For each population x environment combination, the mean, maximum (max.) and minimum values (min.) for the 204 RILs are described for all measured traits. Coefficient of variation (CV, %), heritability (H^2^), and variance components are listed in the table. RIL, recombinant inbred line.

Phenotypic variance of KGR was mostly influenced by genotype and genotype x environment components (Table 2) showing medium (0.61) to high (0.80) heritability in N209xMo17 and R18xMo17 populations, respectively (Table 2). As expected, KW was positively correlated with KGR in both R18xMo17 (r = 0.81, *P* < 0.001) and N209xMo17 (r = 0.55, *P* < 0.001) populations; genotypes with greater KGR showed greater KW.

Genotypic variance of GFD was significant at both populations (*P* < 0.05) but in population N209xMo17 was (13%) lower than the genotype x environment interaction (26%) and residual variances (51%), leading to a low heritability estimate (0.37; Table 2). In population R18xMo17, genotypic variance (21%) was also lower than the residual variance (55%; Table 2). KW was significantly (*P* < 0.001) and positively correlated with GFD for both R18xMo17 (r = 0.60) and N209xMo17 (r = 0.69), genotypes with longer GFD showed greater KW.

KGR is physiologically related to kernel MWC attained at mid-grain filling (Figure 1). For both populations, genotypic variance of MWC was the greatest magnitude component of the phenotypic variance (79 and 64% for R18xMo17 and N209xMo17, respectively), leading to very high heritability estimates (Table 2). KGR was significantly (*P* < 0.0001) and positively correlated with variations in MWC for both R18xMo17 (r = 0.83) and N209xMo17 (r = 0.55) populations.

The duration of the grain filling period depends on how quickly each genotype attains a specific MCPM maturity through the KDR. Estimated residual variance was approximately 60% for both populations, which was greater than genotypic variance (29 and 26% for R18xMo17 and N209xMo17, respectively; Table 2). GFD was negatively correlated with variations in KDR for N209xMo17 (r = −0.46, *P* < 0.001) and R18xMo17 (r = −0.25, *P* < 0.001), respectively.

Similar to KDR, residual variance for MC at physiological maturity was the greatest variance component for this trait, followed by the genotypic, genotype x environment, and environmental variances. Heritability estimates were similar to those for KDR (Table 2). GFD was negatively correlated with MCPM variations in the N209xMo17 (r = −0.41, *P* < 0.001) and R18xMo17 (r = −0.31, *P* < 0.001) populations. Genotypes with shorter GFD reached physiological maturity with greater MC.

In summary, all evaluated traits in the multiparental RIL populations showed significant differences, creating an ample phenotypic variability for QTL detection.

### QTL mapping for individual RIL populations

**Figure 2:**
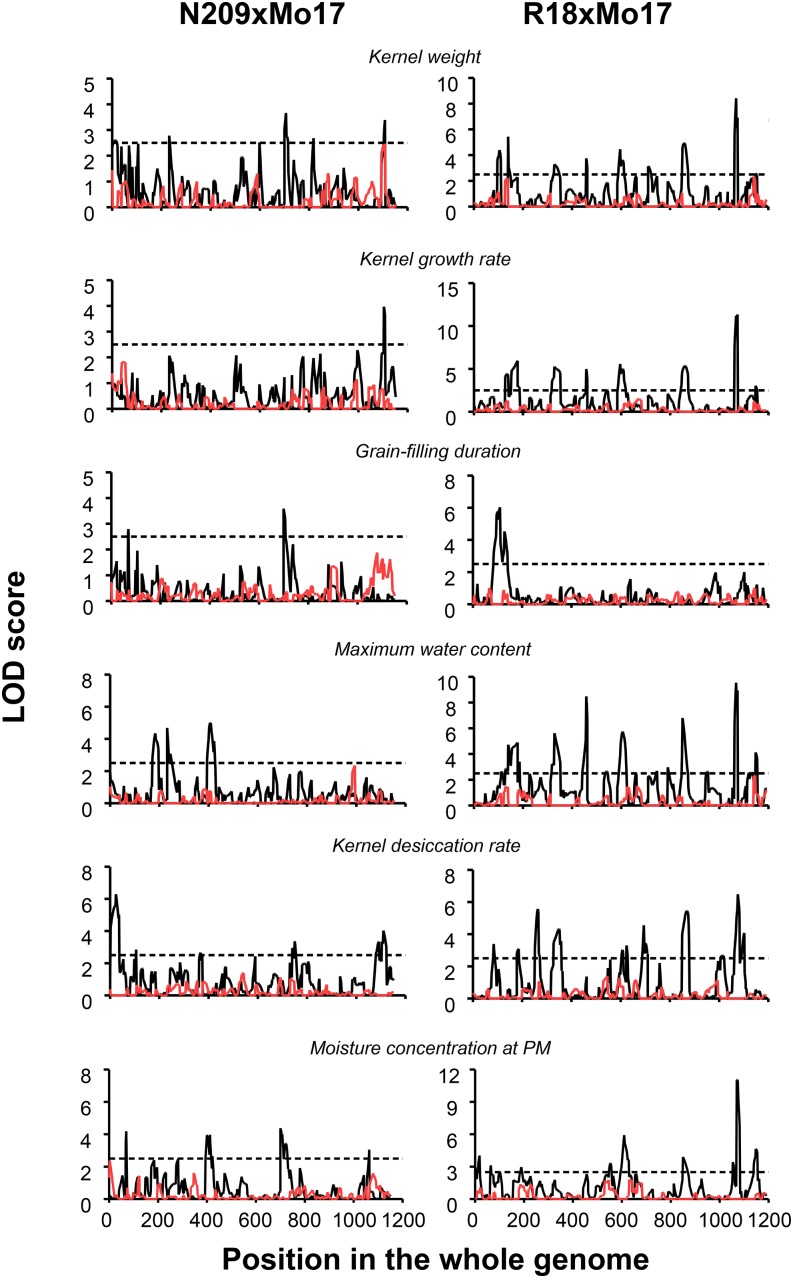
Multienvironmental quantitative trait loci (QTL) mapping results for kernel weight, kernel growth rate, grain-filling duration, maximum water content, kernel desiccation rate, and moisture concentration at physiological maturity for R18xMo17 and N209xMo17 populations, respectively. Black lines represent logarithm of odds (LOD) profile for additive QTL. Red lines represent LOD profile for additive QTL x environment. Dotted lines represent LOD threshold of 2.5.

Individual populations were evaluated under two environments to test for environmentally stable QTL. A total of 23 and 59 environmentally stable QTL were detected along the 10 chromosomes in N209xMo17 and R18xMo17, respectively (Figure 2). The majority of detected QTL were of small effects, except for one QTL for MWC, which showed a relatively major effect (12%) at the N209xMo17 population (Table S1). In population R18xMo17, some relatively major QTL were detected for KGR, KW, MWC and MCPM explaining from 11 to 14% of the phenotypic variance (Table S1).

Several colocalizations were detected among environmentally stable QTL for physiologically related traits. Two QTL for KW in N209xMo17 population colocalized with a QTL for MWC on chromosome 3 and for KGR on chromosome 10 (Figure 2). Both detected QTL for GFD colocalized with QTL for MCPM. In population R18xMo17, all nine detected QTL for KW colocalized with QTL of other grain filling related traits. Seven QTL for KW colocalized with QTL for KGR and MWC. Three and two QTL for KW colocalized with QTL for MCPM and GFD on chromosomes 1, 7, 9, and 10 (Figure 2).

A total of 5 and 17 pairs of markers with significant interaction effects were detected for N209xMo17 and R18xMo17, respectively (Table S2). For N209xMo17 population, all traits except KDR showed one significant epistatic interaction. Among all traits, only one of the five interactions involved loci which both had significant main effects. The remaining four interactions involved loci that were individually nonsignificant (Table S2). Individual epistatic interactions explained up to 5% of the phenotypic variation of KW, 13% for KGR, 10% for GFD, 15% for MWC, and 5% for MCPM (Table S2). For R18xMo17, five pairs of markers showed significant epistatic interactions for KW, two pairs for KGR and KDR, three pairs for MWC, three pairs for GFD, and one pair for MCPM. Among all traits, only two of the 17 interactions involved loci that both had significant main effects. However, seven of the other interactions involved one locus that had a significant effect on the trait. The remaining eight interactions involved loci which were individually nonsignificant. Individual epistatic interactions explained from 3 to 14% of the phenotypic variation of KW, 10% for KGR, from 7 to 11% for GFD, from 5 to 12% for MWC, from 8 to 10% for KDR, and 7% for MCPM (Table S2).

### Joint-population QTL mapping

After testing environmentally stable QTL for individual populations, we tested QTL stability across backgrounds by analyzing both connected populations at two growth environments. A total of 35 QTL were significant for joint multiparental population (Table 3). From total QTL, 16 regions associated with KW (3 QTL), KGR (1 QTL), GFD (2 QTL), MWC (4 QTL), KDR (3 QTL), and MCPM (3 QTL) also were stable across environments showing relatively consistent effects (Table 3).

**Table 3:** QTL for kernel weight, kernel growth rate, grain-filling duration, maximum water content, and moisture concentration at PM identified in the connected RIL populations using joint inclusive composite interval mapping

Trait	Chrom.	Left Marker	Right Marker	Additive Effects 2010		Additive Effects 2011	
				N209xMo17	R18xMo17	N209xMo17	R18xMo17
Kernel weight	1.01	MAIZE.5255.C5	DT272SNP179	13.6	4.7	−	−
	1.05	BG549215	ZM004621	10.0	10.9	7.0	15.9
	1.11	U10418	TC310451	5.8	9.3	−	−
	3.06	TAQKWS4441	AY104231	−	−	7.7	8.3
	5.05	CT08SNP271	ZM011159_18	−17.5	4.9	−14.4	6.1
	9.05	TC306801	CL400916	−	−	4.5	11.0
	10.03	MAIZE.4293.C1	AJ400868	14.1	5.2	13.7	5.9
Kernel growth rate	1.03	OS011512	AY107709	0.016	−0.081	−	−
	1.11	U10418	TC310451	0.003	0.023	0.008	0.017
	5.02	X73980	HHUVIB12	−	−	−0.005	0.017
	9.05	TC306801	CL400916	−	−	0.007	0.021
	10.03	MAIZE.4293.C1	AJ400868	0.019	0.012	−	−
Grain-filling duration	1.05	BG549215	ZM004621	18.0	17.1	24.6	20.1
	7.02	PM0156-620	AY106713	−34.5	9.2	−27.9	7.0
	8.01	AY105770	MAIZE.264.C1	−24.0	14.4	−	−
Maximum water content	3.04	ZM011693	DT134	−11.6	2.7	−12.7	3.0
	4.07	AY104901	BZ534879.1	12.9	6.4	13.8	5.2
	5.02	X73980	HHUVIB12	−	−	3.9	10.9
	5.05	CT08SNP271	ZM011159_18	−9.5	6.5	−6.8	8.4
	9.05	TC306801	CL400916	7.6	8.0	10.2	12.1
Kernel desiccation rate	1.04	AY106901	ZM010356	−0.023	−0.011	−	−
	1.06	AY103580	AY105791_1	−0.022	−0.001	−0.019	−0.004
	2.01-2.02	SM0017D	AZM4_102791	0.011	0.031	0.011	0.022
	5.05	CT08SNP271	ZM011159_18	0.019	0.015	−	−
	7.03-7.04	AY106713	DT188SNP432	0.019	0.005	0.020	0.011
Moisture concentration at PM	1.06	AY105791_1	AY103863	−0.4	1.4	−	−
	2.08	DR791509	AZM4_125066	1.5	0.8	−	−
	4.08	BZ534879.1	BH417806	1.1	1.1	1.2	1.0
	7.02	MAGI_19986_1	ZM004923	2.0	−0.9	1.8	0.4
	9.05	TC306801	AY105818	0.2	1.4	0.8	1.8

QTL, quantitative trait loci; PM, physiological maturity, RIL, recombinant inbred line.

A total of 13 QTL were associated with KW (Table 3) explaining from 12 to 57% of the phenotypic variation across both populations. For both KW components (KGR and GFD) seven and four QTL explaining between 15–24% and 13–46% for KGR and GFD were detected, respectively. Four QTL for KGR and one QTL for GFD colocalized with KW QTL (Table 3). As expected, no QTL colocalization for GFD and KGR was detected (Table 3).

For kernel water related traits 20 QTL were detected along the genome (Table 3) explaining 15–32%, 12–27%, and 12–32% of the phenotypic variation for MWC, KDR, and MCPM, respectively. For MWC, two QTL colocalized with its physiologically related trait KGR, and three QTL colocalized directly with QTL for KW (Table 3). No QTL for MCPM and KDR colocalized with a QTL for GFD (Table 3).

**Figure 3:**
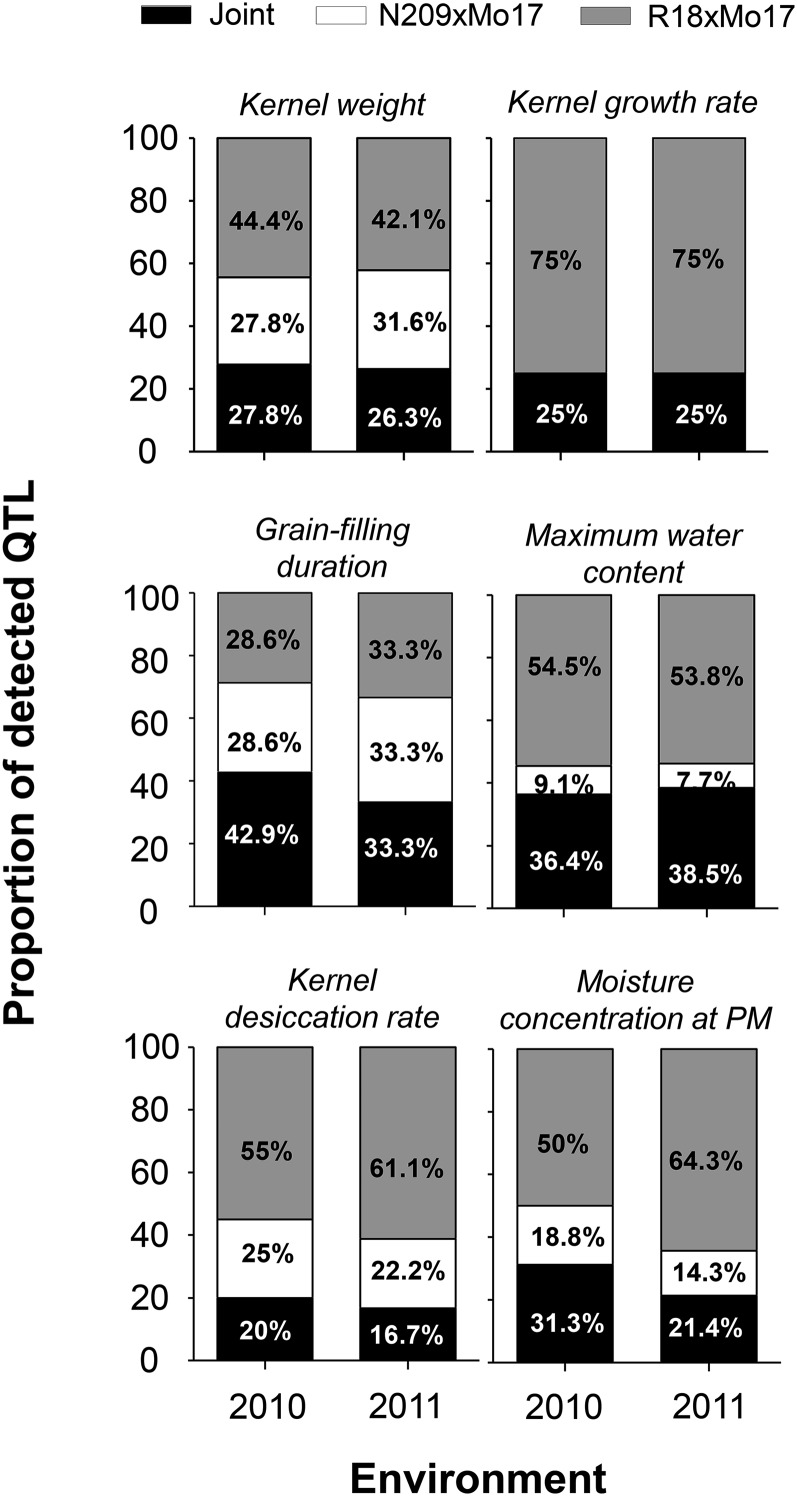
Proportion of detected quantitative trait loci (QTL) in N209xMo17, R18xMo17 and in joint populations for each trait at each environment. Differences between individual and joint population represent the background effects.

When comparing QTL analysis for individual and joint populations, we estimated background effects for each trait at each environment (Figure 3). Between 57 and 83% of detected QTL were population specific, denoting medium-to-high genetic background effects. GFD and MWC were the traits with the lowest genetic background influence, as 43–33% and 36–38% of detected QTL were common for both populations, respectively (Figure 3). KW and KGR showed a greater background effect than GFD, showing 72 to 74% and 75% of the detected QTL to be population specific mostly associated with R18xMo17 population (Figure 3). KDR and MCPM showed the greatest genetic background effects with 80–83%, and 69–79% of the detected QTL being population specific for KDR and MCPM, respectively (Figure 3). As such, the different studied grain filling traits showed QTL results to be more population specific than others.

### Meta QTL analysis

**Figure 4:**
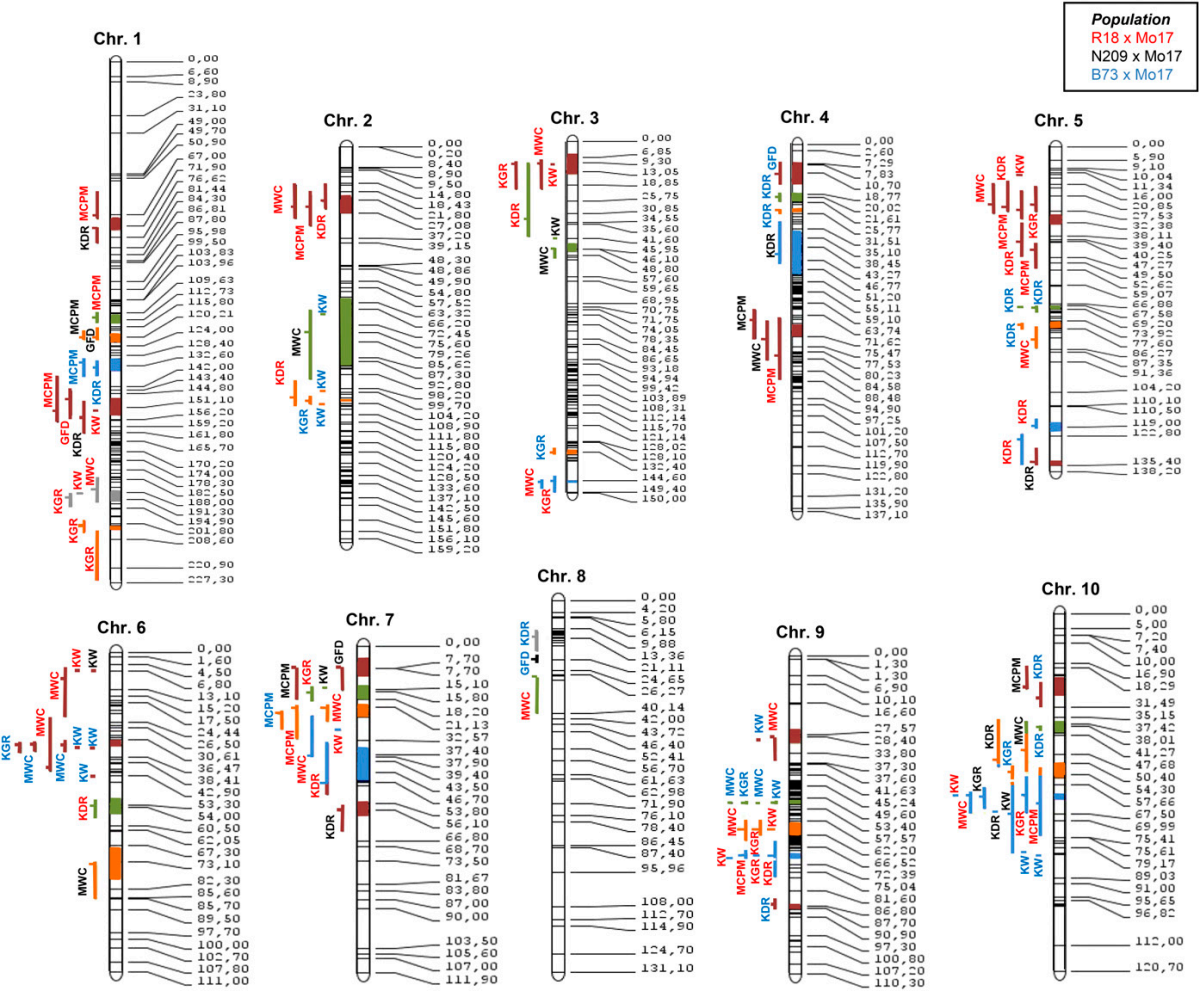
Consensus map including N209xMo17, R18xMo17, and B73xMo17 populations. Meta quantitative trait loci (QTL) are represented with different colors within the chromosomes. Outside each chromosome there are individual QTL from each population. The color of the letters represents the population in which the QTL was detected and bar color the meta QTL to which each individual QTL belongs.

A meta QTL analysis was conducted in order to confirm QTL positions of the evaluated populations. For this we increased background variability using results of environmentally stable QTL for all KW determination traits from the IBMSyn4 (B73xMo17) population from Alvarez Prado *et al.* (2013b). A total of 41 distinct QTL clusters (meta QTL) were identified when considering KW and its physiologically related traits (Figure 4). The number of meta QTL ranged from none in chromosome 8 to seven in chromosome 1. Five meta QTL were considered relevant for further studying for our physiologically correlated traits. Those were located on chromosomes 5, 6, 9 (two meta QTL) and 10 (Figure 4). All relevant meta QTL contained QTL associated with KW, KGR, and MWC, and two of them also showed QTL for MCPM and KDR. Meta QTL from chromosomes 6 and 10 were the most robust, as they contained QTL originally detected at all three populations (Figure 4).

## Discussion

Our study on the genetic basis controlling KW and its physiological mechanisms provided insight in the genetic architecture of an economically relevant trait for maize grain yield determination. The relevance of our study is linking crop physiology mechanisms with genetics using a multiparental RIL population. Previous reports on multiparental RIL populations focused on simpler traits recorded at a fixed stage (Buckler *et al.* 2009; Peiffer *et al.* 2013); however, many traits have a temporal trajectory, and their final value is the consequence of many independent QTL expressed during ontogeny (Maloof 2003). In our study, we dissected a complex trait (KW) in its physiological components where different mechanisms can explain changes in our final trait of interest. We studied the genetic architecture of an emergent trait (KW) through the determination of rate and duration of kernel growth (Figure 1; Yin *et al.* 2004). Specific phenotyping protocols allowed us to precisely characterize a large number of genotypes from contrasting genetic backgrounds for physiological traits related to kernel growth and development.

Complex trait dissection in many species has largely relied on two main approaches, linkage analysis with biparental populations and linkage disequilibrium analysis with association panels. More recently, a maize-nested association mapping population (NAM) was developed combining the favorable properties of both approaches capturing a wide range of maize genetic diversity, exploiting ancestral recombination, and having sufficient power to dissect the genetic architecture of complex traits (Yu *et al.* 2008; Buckler *et al.* 2009; Peiffer *et al.* 2013). In our study we developed a multiparental population following the NAM concept. It was specifically designed for exploring different KW determination strategies and a genome scan with high power for detecting QTL with effects of different sizes (due to differences in parental inbreds). Parental inbreds were previously selected from a grain-filling pattern screening (Borrás *et al.* 2009) and later used for this multiparental population development. This valuable genetic resource showed an ample phenotypic variability, with changes in KW achieved through different combinations of rate and duration of kernel growth.

Results from QTL analysis on our multiparental population confirm previous results using the IBM Syn4 (B73xMo17) population (Alvarez Prado *et al.* 2013b) suggesting that, for maize, the genetic architecture of KW determination is dominated by small additive QTL with several epistatic and medium to low environmental interactions. As expected for complex quantitative traits this study showed evidence of genetic background effects for all grain-filling traits. Background effects are commonly observed across literature in maize (Mihaljevic *et al.* 2004; Li *et al.* 2009) and other plant species (Pilet *et al.* 2001; Xie *et al.* 2008; Zuo *et al.* 2013). According to Holland (2007), inconsistency of QTL across mapping populations is the result of genetic heterogeneity. When many genes altering different physiological mechanisms are controlling a specific trait, different subsets can segregate at different populations (Holland 2007). In the present article, the magnitude of the genetic background affected each grain filling trait differently. When comparing background effects across traits, we found that GFD and MWC were the most stable as the result of changes in the genetic background followed, in descending order, by KW, MCPM, KGR, and KDR. We hypothesized that background effects for our grain-filling traits may ascribe to the physiological complexity of the trait. Thus, traits with a greater hierarchy level (or those that are more complex; like KW) would be more affected than traits with lower hierarchy or are less physiologically complex (all other traits; see Figure 1C); however, our results do not support this hypothesis; KW was among the less affected trait for background effects.

In addition to results from Alvarez Prado *et al.* (2013b) we detected epistatic interactions with minor effects at both individual populations. The finding that epistatic interactions were detected between loci with significant main effects and loci without significant main effects may have implications on the genetic architecture of complex traits (Holland *et al.* 1997). The effects of QTL that exhibit interactions with unlinked genes may be altered dramatically when they are incorporated into a genetic background different from the one in which they were mapped. Our results partially supported this assumption as epistatic interactions were detected among markers for all evaluated traits which showed medium to high background effects, but no association was observed between the number of interactions and the magnitude of background effect. The high number of detected epistasis could be associated with the number of genes involved during the grain-filling period. Liu *et al.* (2008) described 3445 differentially expressed genes associated with processes like cell division, cell growth and differentiation, starch, storage protein, fatty acids, and phytohormone signaling pathways during grain filling.

Consistent colocalization of QTL for MWC, KGR, and KW help explain common phenotypic correlations usually observed (Swank *et al.* 1987; Calderini *et al.* 2000; Borrás and Westgate 2006). QTL colocalization of correlated traits was independent of the genetic background, confirming the common genetic basis previously reported for these traits (Alvarez Prado *et al.* 2013b). These new results confirm our hypothesis that correlated traits are likely to be controlled by the same gene/s.

Evaluated populations showed a high number of stable QTL across environments. This stability could be associated with the large genetic variation respect to genotype x environment interaction variation. Almost 20% of the stable QTL also were stable across genetic backgrounds but showed changes in effect size. When comparing stable QTL with previous results (Alvarez Prado *et al.* 2013b), we confirmed several regions associated with KW determination whereas others remained population specific because of background effects. These relevant regions also were detected in other studies dealing with maize KW (Ribaut *et al.* 1997; Austin and Lee 1998; Melchinger *et al.* 1998; Capelle *et al.* 2010; Peng *et al.* 2011; Guo *et al.* 2011). Although effect sizes may be different, it is important to highlight that several genomic regions were always significant for KW determination (Figure 4), and these may represent regions relevant for identifying genes responsible for natural variation.

KW and its physiologically related traits are relevant for maize grain yield determination. The link between crop physiology and genetics highlighted in this study provided insight into the genetic architecture of KW and the mechanisms involved in its determination. These complex traits are dominated by small additive QTL, several epistatic interactions between significant and nonsignificant main effect QTL, a few environmental interactions, and medium-to-high backgrounds effects. Genetic background effects were significant for all evaluated traits.

Despite several chromosome regions that were stable, effect sizes were modified as the genetic background varied. When comparing results from this study with previous results, we found that several genomic regions were confirmed as stable across environments and genetic backgrounds. Future efforts targeting genes within those regions will help our understanding of maize KW determination.

## Supplementary Material

Supporting Information
