# Supplementary material for: The Genetic Architecture of Maize (Zea mays L.) Kernel Weight Determination
Source: G3 (Bethesda). 2014 Sep 1;4(9):1611–21. doi: 10.1534/g3.114.013243 (PMC4169153; doi:10.1534/g3.114.013243)
Supplement: Supporting Information [file supp_4_9_1611__index.html]

Supporting Information 

# The Genetic Architecture of Maize (*Zea mays* L.) Kernel Weight Determination

## Supporting Information for Prado *et al.*, 2014

**Files in this Data Supplement:**

- File S1 - Raw data (.xls, 1.5 MB)
- Table S1 - Description of significant QTL detected for kernel weight (KW), kernel growth rate (KGR), grain-filling duration (GFD), maximum water content (MWC), kernel desiccation rate (KDR) and moisture concentration at physiological maturity (MCPM) in both individual RIL populations (.zip, 20 KB)
- Table S2 - Description of significant epistatic interactions between markers detected for kernel weight (KW), kernel growth rate (KGR), grain-filling duration (GFD), maximum water content (MWC), kernel desiccation rate (KDR) and moisture concentration at physiological maturity (MCPM) in both individual RIL populations (.zip, 10 KB)
